# Assessment of Zinc Concentration in Random Samples of the Adult Population in Shiraz, Iran

**Published:** 2011-04-01

**Authors:** M H Dabbaghmanesh, H Taheri Boshrooyeh, M R Kalantarhormozi, Gh H Ranjbar Omrani

**Affiliations:** 1 Department of Internal Medicine, Endocrine and Metabolism Research Center, Nemazee Hospital, Shiraz University of Medical Sciences, Shiraz, Iran; 2 Endocrine and Metabolism Research Center, Nemazee Hospital, Shiraz University of Medical Sciences, Shiraz, Iran; 3 Department of Internal Medicine, Boushehr University of Medical Sciences, Boushehr, Iran

**Keywords:** Serum zinc, Deficiency, Prevalence, Adolescent, Iran

## Abstract

**Background:**

Zinc is an essential micronutrient for human health. However, little is known about concentration of this mineral among Iranian population. This study was carried out to determine the current zinc status, evaluate the impact of certain factors like age, sex and Body Mass Index (BMI), and to verify the prevalence of zinc deficiency among Iranian adult population in Shiraz, southern Iran.

**Methods:**

Serum samples from 374 randomly selected healthy individuals living in Shiraz, Iran, aged 19-82 years (143 males, 231 females) were collected and the serum zinc concentration was measured by Flame-Atomic Absorption Spectrometry. We considered the subjects with serum zinc concentration less than 100 μg/dl as zinc deficient.

**Results:**

The serum zinc levels in females were lower than those of males with no statistically significant difference. Serum zinc concentrations were unrelated to age and BMI. It also did not change among different ages and BMI intervals.

**Conclusion:**

About 42.5% of our cases had serum zinc concentration below the cut off value of 100 μg/dl in the serum. Designing appropriate strategies for overcoming this public health problem is necessary.

## Introduction

Zinc (Zn) is a trace mineral important for plants and animals' normal growth and survival as other trace minerals such as iodine, selenium, etc.[1][2][3][4][5][6][7][8][9][10] Zinc is found in all parts of human body including organs, tissues, bones, fluids, and cells. Because zinc is used to generate cells, it is especially important during pregnancy for growing the fetus whose cells are rapidly dividing.[2][11][12] Moreover it is vital to activate the growth (height, weight and bone development) in infants, children and teenagers.[2]Zinc has been proven to be effective against infections.[2][13][14] Among all the vitamins and minerals, zinc shows the strongest effect on the immune system.[2][3] An estimated 3000 proteins in the human body contain zinc prosthetic groups. The most-important types of protein containing zinc are enzymes and transcription factors such as metalloenzyme and zinc finger.[6][10] Zinc-containing enzymes are used by the body to regulate growth and development, promote fertility, and help digestion and nucleic acid synthesis.[12]

Zinc deficieney occurs where insufficient zinc is available for metabolic needs. It is usually nutritional but it may be caused by malabsorption, acrodermatitis, diabetes, malignancies, and other chronic illnesses.[1][4][12] Animal products, such as shelfish and red meat which contain substantial amounts of zinc in readily absorbable form, are not consumed extensively in many parts of the world because of their high cost and limited supply.[1][15] On the other hand, consumption of the diets based on plant foods, especially those diets rich in phytate which is a potent inhibitor of zinc absorption, has resulted in zinc deficiency.[1][12] Thus, many people- especially in lowincome families in developing countries- are unlikely to receive adequate zinc from their diets.[1][4]

In a report in 2002, the World Health Organization (WHO) measured the amount of disease, disability and death which can be attributed to major health risks. Zinc deficiency was shown to be one of the leading causes of illnesses and diseases in low income countries. In developing countries, zinc deficiency ranked the 5th among the leading 10 risk factors. WHO attributes 800,000 deaths worldwide each year due to zinc deficiency.[1]

Zinc deficiency has serious consequences for health, including impairment of the immune system and as a result increased prevalence of childhood infections such as diarrhea and pneumonia, impaired growth and development of infants, children and adolescents, and impaired maternal health and pregnancy outcome.[1][12][16]

Regarding the importance of zinc in the health of the individuals, different studies have been performed in various parts of the world to measure serum zinc concentration, the impact of several factors such as sex, age, Body Mass Index (BMI), smoking and drinking habits, and socioeconomic status on it.[17][21]

Zinc deficiency is associated with disease conditions and our country, Iran, is located in an area with a high prevalence of zinc deficiency due to a low dietary intake of zinc-rich foods such as animal-source ones in which zinc is more bioavailable, and high consumption of legumes and cereals, which retain inhibitors of zinc absorption. To the authors΄ knowledge, the present study is the first one that was performed on healthy adult population in the south of Iran (in Shiraz, center of Fars province). Although a few studies have been performed to evaluate the zinc concentrations in limited age intervals, they have not focused on both sexes. Therefore, we intended to determine the serum zinc concentration, evaluate the influence of certain factors like age, sex and BMI, and to verify the prevalence of zinc deficiency in adult population in the city of Shiraz in southern Iran.

## Material and Methods

This analytical cross-sectional study was conducted in Endocrine Research Center at Shiraz University of Medical Sciences in 2007 in Shiraz, Iran. The study protocol was approved by Reviewer Board of Shiraz Endocrine Research Center and Shiraz University of Medical Sciences Ethics Committee.

According to the results of previous studies, sample size for the estimation of mean serum zinc concentration was calculated to be 362 subjects using the following formula: n=[z×s/d]2 where n=required sample size, z=value for selected alpha level of 0.025 in each tail=1.96, s=estimate of standard deviation and d=acceptable margin of error for mean.

The subjects were selected, using random sampling from the 8 areas of Shiraz city.

First, postal codes that terminated in even digits from 8 areas of Shiraz city were chosen. Then, from each area, 50 individuals were selected (50 families from each area and one person from each family) through the table of random numbers. A written informed consent form was signed by each subject after explaining the nature of the study.

The exclusion criteria were the presence of gastrointestinal and hepatic disorders, renal and cardiovascular diseases, cancerous diseases, smoking, and use of oral contraceptives, pregnancy and a vegetarian diet. After excluding the participants with aforementioned conditions, total number of the subjects that participated in our study reached 374 cases.

Anthropometric measurements were done for participants wearing light clothing without shoes by a trained research assistant. The weights of all participants were measured to the nearest 0.1 kg with a portable digital balance. The heights of all participants were measured to the nearest 1 cm with a portable stadiometer. BMI was calculated as: weight (kilogram)/ height² (meter). BMI was classified into four groups: BMI<20, 20≤ BMI<25, 25≤BMI<30, BMI≥30. The measurement of serum zinc was performed by atomic absorption spectrometry (variant Chemthech Analytical 2000). Zinc concentration more than 100 μg/dl was considered as normal values.[22][23]

Statistical analyses were performed with the Statistical Package for Social Sciences (SPSS, version 16, Chicago, IL, USA), and statistical methods used in this study were described in the manual included in the software.[24] The data was presented as mean (±SD). One way ANOVA and Student´s two tailed t-test were used to compare the mean values obtained in different groups. Comparisons of frequencies were made by Chi Square test. Pearson correlation coefficient was used to study the correlation between zinc and age and BMI. In all analyses, the level of significance was considered as p<0.05.

## Results

After interview and completion of the related questionnaires, 374 participants entered the survey (143 men and 231 women) with an age of 38±13.37 years (range between 19-82 years) and BMI of 25.37±4.55 kg/m2. The mean serum zinc concentration obtained in this study and those from other published studies for other population are presented in Table 1.

**Table 1:** The mean serum zinc concentrations in this and other published studies.

**Zn (µg/d)**	**No.^k^**	**Reference No^l^**	**Country**
103.66 ±18.06 a	374	this study	Iran
100±10^b^	372	25	Sweden
116 ±52^c^	395	26	Spain
113.9^d^	434	18	Spain
97.22^e^	186	28	Spain
91±27^f^	1155	29	Csech
72.2^g^	44	30	Bangladesh
75.36^h^	102	31	Iran
95.2±17^i^	881	32	Iran
104^j^	420	27	Iran

^a^ Values for 19 to 82 years old subjects

^b^ Values for 15 years old subjects

^c^ Values for 6 to 75 years old subjects

^d^ Not given

^e^ Values for 18 to 65 years old subjects

^f^ Values for 6 to 65 years old subjects

^g^ Values for 22 to 28 years old males

^h^ Mean age 33.8 ±10.7 years

^i^ values for 11 to 16 years old subjects

^j^ Mean age 18.87±1.31 years females

^k^ Number of participants in the study

^l^ See references

**Fig. 1:**
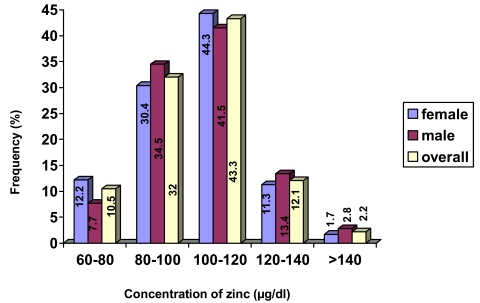
Distribution of serum zinc concentrations for overall and both groups of males and females. Each bar represents the frequency of individuals that have the specific value of concentration of zinc (overall and by sex). The concentration of zinc was divided into five groups that was shown on horizontal axis.

**Fig. 2:**
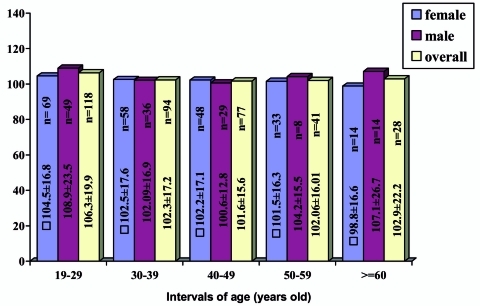
Serum zinc concentrations for all samples, males and females grouped by intervals of age. Each bar represents the mean±SD value of zinc concentration and number of individuals by an age specific group.

Table 2 shows the results of serum zinc concentration grouped according to sex, age and BMI. The data for zinc ranged between 68.4 and 205 μg/dl, presenting a mean zinc concentration of 103.66±18.06 μg/dl. Serum zinc levels in females were lower than those in males without a statistically significant difference (p=0.268). Moreover, this value did not change among different BMI groups (p=0.941). The distribution frequencies of zinc concentrations according to gender are shown in Figure 1. Most of the analyzed samples (57.5% of the total) had zinc concentrations above 100 μg/dl. (57.7% of males and 57.4% of females). The evolution of the serum zinc concentrations with the age of the individual in all the samples and grouping by gender is shown in Figure 2.

**Table 2:** Results of the serum zinc concentration of the Iranian population by sex, age and BMI.

**Variable**	**Zn (µg/dl)**			
	**No.**	**X±SD**	**Minimum**	**Maximum**
Overall	374	103.6±18.06	68.4	205
Sex				
Male	143	105.05±19.6	76	205
Female	231	102.9±16.9	68.4	163.3
		P^a^=0.268		
Age				
19 to ≤29	118	106.3±19.9	68.4	205
30 to ≤39	94	102.3±17.2	69.7	149.3
40 to ≤49	77	101.6±15.6	71.4	149.9
50 to ≤59	41	102.06±16.01	73	132.8
≥60	28	102.9±22.2	72.8	187.5
		P^b^=0.36		
BMI (kg/m^2^)				
<20	40	102.9±12.3	75.1	121.3
20-24.9	147	103.7±19.4	71.4	205
25-29.9	122	104.5±18.01	69	175.9
≥30	50	103.06±18.7	68.4	149.9
		P^b^=0.941		

^a^ For comparison of the mean serum zinc concentration between sexes, independent sample t test was used

^b^ For comparison of the mean serum zinc concentration between different age and BMI groups, One Way Analysis of Variance was used

The mean zinc concentration in the serum did not significantly change among different age intervals (p=0.36). For females, the mean zinc concentration in the serum showed a decreasing trend with an increase in age interval but without any significant differences among groups (p= 0.787). Thus, the females in group of more than 60 years old had the lowest value in the serum zinc concentration.

For males, the lowest and highest mean zinc concentration was observed in the interval age of 40-49 and 19-29 years old, respectively. However, there were no significant differences among groups (p=0.380). Serum zinc concentrations were unrelated to age (r=- .0.093, p=0.079) and BMI (r=-0.033, p=0.532). The zinc deficiency prevalence based on having serum zinc concentration less than 100 μg/dl was 42.5% [confidence interval (CI): 37.5-47.4% with 95% confidence]. The deficiency prevalence in females (42.6%; CI: 36.2-48.9%) was higher than that in males (42.3%; CI: 34.1-50.4%) with no significant difference (p=1). This prevalence in the 40-49 year old age group (49.3%) was higher than the others (Table 3). Zinc deficiency prevalence among five age intervals in the total cases (p=0.23) and also between males and females in each age group was not significantly different (Table 3). Moreover, comparison of this value among four BMI groups indicated no significant difference (p=0.778).

**Table 3:** Frequency distribution of zinc deficiency prevalence for the population classified by age group.

	**19-29 years**		**30-39 years**		**40-49 years**		**50-59 years**		**≥60 years**	
	**Deficient a**	**Nl b**	**Deficient**	**Nl**	**Deficient**	**Nl**	**Deficient**	**Nl**	**Deficient**	**Nl**
Male	32.7	67.3	52.8	42.7	51.7	48.3	50	50	35.7	64.3
Female	37.7	62.3	44.8	55.2	47.9	52.1	45.5	54.5	50	50
	P^c^=0.697		P^c^=0.526		P^c^=0.816		P^c^=1		P^c^=0.704	
Total	35.5	64.5	47.8	52.2	49.3	50.7	46.3	53.7	42.8	57.2

^a^ Values are presented as percentile

^b^ means normal

^c^ For comparison of frequencies between sexes, Chi-Square test was used. No significant difference was shown in zinc deficiency between males and females by age group

## Discussion

The findings of our study showed that the mean serum zinc concentration was 103.66±18.06 μg/dl, being lower in females than in males without statistically significant difference. Serum zinc concentrations were unrelated to age and BMI. This value did not change among different ages and BMI intervals. Also, the results of this study provided evidence for the existence of zinc deficiency (42.5%) among adults in Shiraz city. This prevalence in females was higher than that in males with no statistically significant difference. Also, comparison of this value among BMI and age intervals indicated no statistically significant differences (p=0.778, p=0.293, respectively).

The mean serum zinc concentrations in our study are in agreement with the results of some studies.[18][25][26][27]

On the other hand, in some studies, this value was lower than that of the present study.[28][29][30][31][32] In our study, females showed lower zinc levels than males but there were no statistically significant differences which agrees with most of the findings of other studies. [26][28][31][33] However, the results of two studies in Thailand and Spain showed that the mean serum zinc concentration in females was higher than that in males without and with significant differences, respectively.[18][34] Also, the mean zinc concentration in the serum did not significantly change among different age intervals considered. This is in the same line with the findings of some other studies.[26][31][35]

Declining mean serum zinc concentration was observed with the increase of age intervals among females in our study without any significant differences, being consistent with the findings of another study.[36] No relationship was observed between values of BMI and zinc concentrations in this study. Such a finding is similar to that of some research in this field,[31][37] and also in contrast with that of another study.[38]

The results of this study provided evidence for the existence of zinc deficiency among adults in Shiraz city. In our study, the zinc deficiency prevalence was 42.5%, being 42.6% in females and 42.3% in males with no significant difference. In one study conducted on students with an age interval of 11-16 years in Iran in 1997, the prevalence of zinc deficiency was 31%.[32]

In another study with a cut off point less than 70 μg/dl for zinc deficiency, this value was 47.1% (37.29% in males, 60.47% in females).[31] Furthermore, in another study performed on Iranian females in 2005 with a mean age of 18.87 years, this value was reported 7.1% (with a cut off point less than 85 μg/dl).[27] As observed, in the aforementioned studies, different values were reported for zinc deficiency among the studied population and selecting different cut off points for determining the prevalence of zinc deficiency that could be a reason for this variation. Other reasons could be the method of sampling, age of the studied population and the geographical region where the study was performed. In the present study, the prevalence of zinc deficiency among females was higher than males with no statistically significant difference. Hashemi et al. in their study came to the same conclusion.[31] However, the results of another study are in contrast with ours.[32]

Zinc as a trace element has an important role in human health and its deficiency results in undesirable effects in functioning of different bodily organs. Moreover, according to WHO report, the prevalence of zinc deficiency in Eastern Mediterranean Regional Office (EMRO) (our country, Iran was located in this region) is 25-52%. Therefore, it seems that the use of methods such as food fortification which is the addition of nutrients to commonly eaten foods, beverages or condiments,[1][12][13] strategies to modify or use a variety of diets in order to improve access to foods with a high level of absorbable zinc,[1][12] and genetic modification of plants to increase their level of absorbable zinc,[1][13] are recommended for eliminating zinc deficiency. Other alternatives are implication of zinc supplementation especially for high risk populations,[12][13][39][40] household intervention for increasing the zinc content, the reducing of the phytate content of diets, i.e. germination to increase phytase activity, fermentation to increase microbial phytase activity, and soaking to reduce phytic acid content, and finally using natural and artificial fertilizers in areas where zinc content of the soil is very low.[12][13] Furthermore, it seems that comprehensive studies are required to be designed to determine a precise cut off point for estimation of zinc deficiency prevalence in our country so that an appropriate guidance can be established for this issue.
